# Case Report: Long-Term Tolvaptan Treatment in a Child With SIADH and Suprasellar Arachnoid Cyst

**DOI:** 10.3389/fped.2021.684131

**Published:** 2021-07-16

**Authors:** Andrea Puma, Milena Brugnara, Paolo Cavarzere, Marco Zaffanello, Giorgio Piacentini, Rossella Gaudino

**Affiliations:** ^1^Department of Surgical Sciences, Dentistry, Gynecology and Pediatrics, Pediatric Division, University of Verona, Verona, Italy; ^2^Department of Surgical Sciences, Dentistry, Gynecology and Pediatrics, Pediatric Nephrology Division, University of Verona, Verona, Italy; ^3^Department of Surgical Sciences, Dentistry, Gynecology and Pediatrics, Pediatric Endocrinology Division, University of Verona, Verona, Italy

**Keywords:** suprasellar arachnoid cysts, syndrome of inappropriate antidiuresis, hyponatriemia, vaptans, tolvaptan, pediatric treatment

## Abstract

Suprasellar arachnoid cysts represent a rare occurrence in the pediatric population and usually cause symptoms related to mass effect and can occasionally cause endocrine dysfunctions. The association between SAC and the syndrome of inappropriate antidiuretic hormone (SIADH) in the pediatric population has rarely been described previously. In most cases, SIADH is temporary and resolves by treating the underlying cause. The first-line treatment consists of fluid restriction in asymptomatic children. Oral urea and demeclocycline are other effective treatment options. Vaptans are a new class of medication for the management of SIADH. These agents are a nonpeptide vasopressin V2 receptor antagonist that selectively antagonizes the antidiuretic effect of AVP, resulting in excretion of diluted urine or “aquaresis.” Their efficacy has been shown in adult patients with euvolemic or hypervolemic hyponatremia. However, evidence is lacking in pediatric patients with SIADH. We report the case of a 9-year-old female child with a SAC, who underwent endoscopic fenestration at the age of 2 years. After surgery she developed chronic hyponatremia due to SIADH. Hyponatremia was refractory to treatment with fluid restriction, oral sodium, and urea. In order to normalize serum sodium levels, tolvaptan treatment was started on a compassionate-use basis; 24–48 h later serum sodium levels returned to normal. To date, tolvaptan has been used regularly for 6 years with no side effects occurring during the treatment period. This is the first case of a child with chronic SIADH secondary to SAC successfully treated with tolvaptan. Further studies are needed to demonstrate its usefulness on a broader case series.

## Introduction

Suprasellar arachnoid cysts (SAC) represent a rare occurrence in the pediatric population (1–3). SACs usually cause symptoms related to mass effect leading to macrocrania and obstructive hydrocephalus, but occasionally they cause visual and/or endocrine dysfunctions. Precocious puberty and growth hormone deficiency are the most common endocrine conditions reported (4–6).

SIADH consists of hypotonic hyponatremia, in a euvolemic state, caused by impaired free water excretion resulting from nonphysiological stimuli for arginina vasopressin (AVP) production in the absence of renal or endocrine dysfunction. SIADH is most often the consequence of central nervous system (CNS) disorders, pulmonary disorders, malignacies and medication (7).

First-line treatment of SIADH consists of fluid restriction in asymptomatic children, while infusion of hyperosmolar saline is required in severe cases with neurological symptoms. In most cases, SIADH is temporary and resolves by treating the underlying cause (8). Oral urea and demeclocycline are other effective treatment options suggested in the literature (9).

Vaptans are a relatively new class of medication for the management of SIADH. These agents represents a nonpeptide vasopressin V2 receptor antagonist that selectively antagonizes the antidiuretic effect of AVP, resulting in excretion of diluted urine or “aquaresis” (10). Their efficacy has been demonstrated in adult patients with euvolemic or hypervolemic hyponatremia. However, evidence in pediatric patients with SIADH is lacking, and, to date, there is only one ongoing trial registered in Europe that includes children and adolescents. Tolvaptan is the only vaptan approved by the European Medicines Agency for the treatment of hyponatremia due to SIADH (10).

The association between SAC and SIADH in the pediatric population has rarely been described previously (11). We report for the first time, the case of a child with a SAC who underwent brain surgery, resulting in chronic severe hyponatriemia due to SIADH. She was treated with tolvaptan for 6 years.

## Case Description

**Figure 1:**
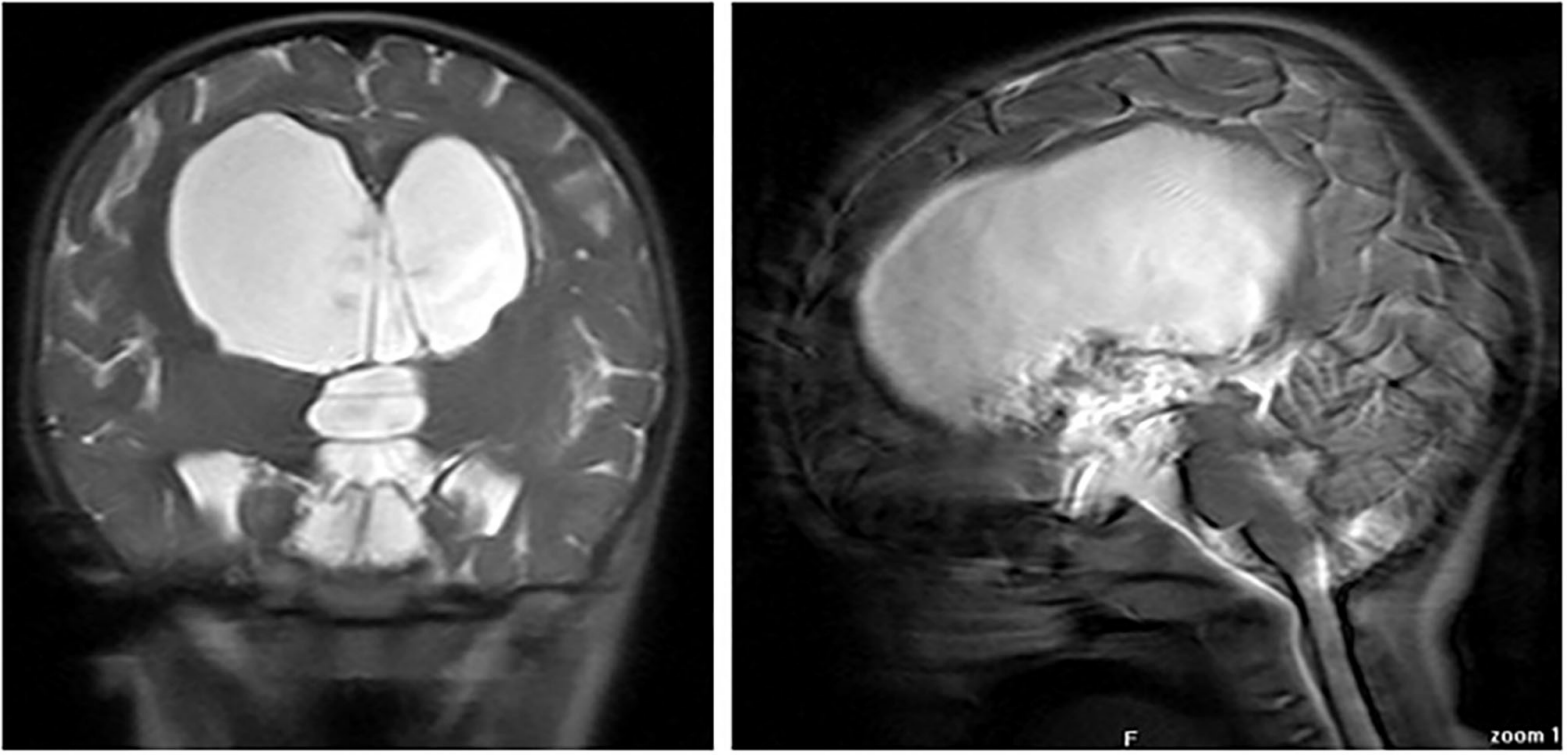
Coronal (right) and sagittal (left) MR images at diagnosis, showing large suprasellar arachnoid cyst.

A 2-year-old female infant was admitted to hospital with progressively increasing head circumference and neuro-psycomotor development delay after the age of 8 months. An eye fundus examination revealed papilloedema and brain magnetic resonance imaging (MRI) showed a large suprasellar arachnoid cyst (SAC) with signs of hypertensive hydrocephalus (Figure 1). Plasma sodium was 132 mmol/L. Hence, she underwent a neurosurgical endoscopic cystostomy.

A follow-up brain MRI 6 months later showed only a minor reduction of the SAC and lab tests revealed plasma sodium of 128 mmol/L. Another surgical procedure of cyst fenestration was scheduled. A post-op MRI/CT scan revealed a small reduction of the cyst.

At the age of 3 years, during non-febrile acute gastroenteritis, she had a seizure; the acute episode was treated with rectal diazepam. After she was admitted to our emergency department intravenous isotonic saline solution was started while waiting for the results of blood tests. Laboratory evaluation revealed hyponatremia (serum sodium level 123 mEq/L), with normal findings for blood gas analyses, electrolytes, and renal function tests. For symptomatic hyponatremia, a modest increase in the serum sodium level was obtained through the administration of 3% salts, 2 mL/kg delivered as bolus. Physical examination revealed body weight stable at 15.7 kg (50th−75th percentiles) and did not show any signs of dehydration nor oedema. Serum osmolality was decreased (267 mOsm/L), whereas urine osmolality had increased (725 mOsm/L). Urinary sodium concentration was increased to 227 mEq/L. The results of the laboratory evaluation at diagnosis are shown in Table 1. An electroencephalogram was performed and showed non-specific alterations. Clinical and laboratory findings such as euvolemic hyponatremia, decreased blood uric acid concentration, low plasma blood urea nitrogen (BUN < 10 mg/dl), normal serum creatinine, low plasma renin activity, aldosterone, and serum hypo-osmolality with increased urine osmolality led us to diagnosis of SIADH. Extensive investigation was performed to understand the etiology of SIADH; thyroid function test, and serum concentrations of adrenocorticotropic hormone (ACTH), cortisol, renin, and aldosterone were normal. For these reasons we concluded that SIADH was caused by the intracranial arachnoid cyst. The patient Body Surface Area (BSA) was 0.66/m^2^, and his fluid requirement (calculated with Holliday–Segard Normogram) was 1,285 ml. Treatment was started with water restriction (1,200 ml/m^2^ of BSA equivalent to 62% of his fluid requirement) and salt supplement (oral NaCl 3.8 mEq/kg/day). NaCl oral supplementation was initially determined with formula: desired Na (135)–[Na^+^] × Total Body Water (weight kg × 0.6).

**Table 1:** Patient's laboratory values, obtained after initial correction of 3% salts, 2 mL/kg delivered as bolus, at SIADH diagnosis.

**Laboratory values**	**At diagnosis**	**Normal values**
Plasma osmolality (mOsm/kg)	267	275–295
Urine osmolality (mOsm/kg)	725	50–1,200
P–Na (mmol/L)	128	135–145
P–K (mmol/L)	4.21	3.4–4.8
P–C l (mmol/L	97	95–107
P–Ca (mg/dL)	8.81	8.4–10.4
U–Na (mmol/L)	227	<20
Urea (mg/dL)	15.3	11–39
Creatinine (mg/dL)	0.3	0.5–1.2
ACTH (pg/mL)	12.4	8.5–50
Cortisol (mcg/dL)	26.2	4–25
Renin (pg/mL)	2.28	1.98–24.6
Aldosterone (pg/mL)	147	35–300
U-ADH (pmol/L)	43	

An endocrinological follow-up was scheduled to evaluate regulation of water balance and hypothalamic antidiuretic hormone (ADH) release, her ongoing urine osmolality was 266 mOsm/kg. The water restriction, salt and urea supplementation have been estimated on the basis of levels of urinary and plasma electrolytes, her fluid requirements and her ongoing urine osmolality (that's always been <600 mOsm/kg.). Further water restriction (1,000 ml/m^2^ of BSA equivalent to 51% of his parental fluid requirements), salt supplement (oral NaCl 5 mEq/kg/day) and 30% oral urea treatment (started at 0.1 g/kg/day and increased gradually to 0.4 g/kg/day and stopped for the poor compliance) were not sufficient to maintain serum sodium at acceptable levels. Levels remained unstable, ranging from 126 to 130 mmol/L. Fluid restriction was difficult to maintain; salt supplementation and oral urea, which had a bad taste, were poorly accepted by the child. For these reasons, we decided to start an oral low-dose treatment with tolvaptan at 3.75 mg, increasing to 7.5 mg. Tolvaptan was started within the recovery regimen with close monitoring of oral fluid intake and urine output, as well as Na, K, and Cl concentrations. Following initiation of tolvaptan, water restriction was discontinued and the patient's family was educated on the potential effects of tolvaptan therapy.

At the age of 8 years, the girl showed central early puberty and GHRH-analog treatment was started for psychological immaturity and short stature. GH deficiency was diagnosed at the age of 9 years and she began recombinant GH therapy.

**Figure 2:**
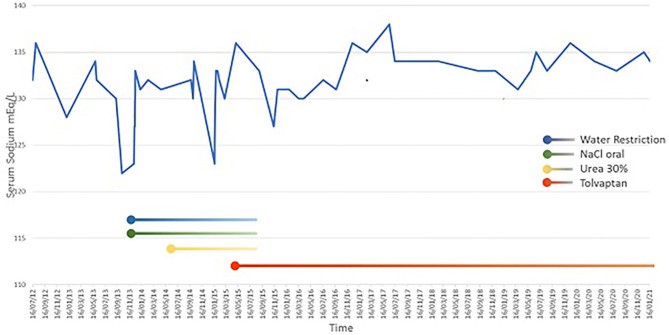
Serum sodium values during different treatments.

Now, 6 years after starting therapy with tolvaptan, serum sodium levels are nearly normal, ranging from 133 to 136 mmol/L. Tolvaptan dosage is 11.25 mg/day and no severe hyponatremia has been noted (Figure 2). There have been no side effects during treatment.

## Discussion

We report the case of a minor with SAC, surgically treated, with symptomatic chronic hyponatremia due to SIADH. For the first time, our case described the use of tolvaptan in SIADH due to a SAC referred for brain surgery. However, no adverse events were found in long-term treatment.

Suprasellar arachnoid cysts (SACs) constitute 5–12% of intracranial arachnoid cysts (12). On review of several studies, the typical patient with a SAC likely presents with macrocrania, obstructive hydrocephalus, psychomotor retardation, incidental discovery of the cyst prenatally, headache, nausea and vomiting, seizures, decreased visual acuity, endocrine disorders, head bobbing, and neurological deficits due to mass effect (4–6, 13).

SACs are a well-known cause of hypothalamic-pituitary disfunction due to their anatomical location (14), and a wide spectrum of endocrine disorders can be found in patients with SAC, ranging from isolated hormone abnormalities to panhypopituarism associated with diabetes insipidus (6).

Long-term follow-up results of patients with SAC showed that endocrine disorders can develop even years after the initial diagnosis of the cyst; and new endocrine abnormalities were detected in both operated and non-operated patients. No significant associations were found between surgical treatment and endocrine outcome (14).

Our patient manifested SIADH at 3 years of age, early puberty at 8 years, and GH deficiency at 9 years.

To date, there is only one other case in the literature of a SAC associated with SIADH. Beier et al. reported the case of an infant that was diagnosed prenatally with a SAC. At the age of 5 months the patient developed hydrocephalus due to cyst enlargement and the baby was scheduled for surgery. However, preoperative lab tests revealed SIADH. The patient underwent endoscopic cyst fenestration after stabilizing serum sodium concentrations with fluid restriction and administration of 3% sodium chloride. Postoperatively, she had complete resolution of SIADH (11).

In our case, when the child was ill and had a seizure, and considering her neurosurgical history, she was immediately tested for hyponatremia, but intravenous isotonic fluids were started pending the results of the tests.

The volume status is hard to assess accurately and there are no specific biochemical tests for diagnosing SIADH, so diagnosis may be challenging. Most seriously ill children are at risk of developing hyponatremia due to numerous physiological stimuli for AVP production and to the disease states associated with SIADH. In this setting, administration of hypotonic fluids is the most important factor contributing to the development of hyponatremia. For this reason, it should be kept in mind that patients with conditions associated with SIADH risk developing severe hyponatremia, and hypotonic maintenance fluids should be avoided in these patients (15). Proper diagnosis of this disorder is crucial for successful treatment. SIADH is essentially a diagnosis of exclusion, so it may be challenging. Most hyponatremic patients who appear to be euvolemic by physical examination have the SIADH. However, such patients may occasionally have hyponatremia due to true volume depletion; the volume status is hard to assess accurately and there are no specific biochemical test for determining the effective circulating volume. In our patient, the laboratory results (low plasma blood urea, normal serum creatinine, and low renin activity) support that this child presented with euvolemic hyponatremia, a key factor in making a diagnosis of SIADH. Moreover, children with euvolemic hyponatremia should be checked for serum concentrations of thyroid stimulating hormone (TSH), thyroid hormones, adrenocorticotropic hormone (ACTH), cortisol and renin because of severe hypothyroidism, and cortisol deficiency can mimic SIADH (15).

SIADH did not resolve in our patient after the seizure, but it evolved to a chronic form.

The first-line treatment of SIADH is fluid restriction, although there are no clear guidelines regarding the exact amount of fluid restriction required. The maximal fluid restriction in an adult with an otherwise normal renal solute load is generally considered to be about 1,000 mL/day (16). However, this level of water restriction is quite difficult to achieve in infants as their nutrition mainly consists of milk and fluids, and these patients have a lowered osmotic threshold for thirst (17). For patients who do not respond to fluid restriction, the next step for children would be to increase oral sodium intake or give oral urea to increase the renal solute load, thereby inducing osmotic diuresis. Oral urea has proven to be successful in treating chronic hyponatremia in both children and adults who did not respond to conservative measures (9, 18) but it was hardly ever used in children because of its bad taste, even if, a more palatable formulation of oral urea is recently available (i.e., Ure-Na^TM^).

In our patient, fluid restriction, salt supplementation and oral urea were ineffective in maintaining acceptable serum sodium levels; and owing to the severity of symptoms related to the hyponatremic condition, we chose to start an off-label medicine called tolvaptan. That resulted in normalization of serum sodium levels, allowing us to stop water restriction and salt supplementation and to liberalize fluid intake.

There have been numerous placebo-controlled trials which have demonstrated the safety and efficacy of tolvaptan for the treatment of hyponatremia associated with SIADH in adults (19, 20).

The European Medicines Agency has approved tolvaptan for the treatment of hyponatremia due to the syndrome of inappropriate antidiuretic hormone secretion (SIADH) in adults, whereas the United States Food and Drug Administration recommends tolvaptan for the treatment of both euvolemic and hypervolemic hyponatremia in adults. To date, there is limited information and few data available regarding tolvaptan use in children (10).

The use of tolvaptan in childhood has been reported in children with hypervolemic hyponatremia due to heart failure and nephrotic syndrome. Some studies on the use of tolvaptan in children with hypervolemic hyponatremia are reported in Table 2A (21–24).

**Table 2A:** Main studies on tolvaptan use in children with hypervolemic hyponatremia.

**References**	**Study design**	***N***	**Median age**	**Median tolvaptan dose**	**Dosing period**	**Adverse events**
Regen et al. (21)	Single-center, retrospective study	28	2 years (1 month-18 years)	0.3 mg/kg (range 0.1–1.3 mg/kg)	3 days	None
Higashi et al. (22)	Multicenter, observational study	34	1 year (2–202 months)	0.25 mg/kg (range 0.02–0.76 mg/kg)	30 days	Thirst and dry month (six patients)
						Mild increase in ALT/AST (one patient)
Katayama et al. (23)	Single-center, retrospective study	25	26.1 ± 32.4 months	0.45 mg/kg	Single dose of TLV (1 day)	None
Kerling et al. (24)	Single-center, retrospective study	25	Responder: 35 days (9–228)	Responder: 0.53 mg/kg (0.15–1.06 mg/kg)	TLV Responder: 8 days (1–25 days)	One event in the TLV non-responder group: hypernatremia (151 mmol/l) on 9th day
			Non-responder: 37.5 days (20–549)	Non-responder: 0.49 mg/kg (0.13–0.95 mg/kg)	TLV Non-responder: 7 days (1–47 days)	

There is currently only one ongoing study to assess tolvaptan's effect on fluid balance in children and adolescent subjects with euvolemic or hypervolemic hyponatremia.

Recently there have been a few reports on the use of tolvaptan as treatment for SIADH in infancy; but to date, experience is limited. In Table 2B, we have summarized the characteristics of the seven cases described in the literature (25–29).

**Table 2B:** Characteristics of the 7 children with SIADH treated with tolvaptan described in the literature.

**Reference**	**Study design**	***N***	**Median age**	**Median tolvaptan dose**	**Dosing period**	**Adverse events**
Marx-Berger et al. (25)	Case series	2	4 months	0.4 mg/kg/die	7 months	None
Willemsen et al. (26)	Case report	1	11 years	0.28 mg/kg/die	1 month	Desquamative rash on hands)
Tuli et al. (27)	Case series	3	6 years	0.2 mg/kg/die	Maximum 4 years	None
Koksoy et al. (28)	Case report	1	16 years	0.14 mg/kg/die on alternate days	2 weeks	None

As in the aforementioned reports, also in our case treatment was started within the recovery regimen and under hospital conditions with close monitoring of oral fluid intake and urine output, as well as Na, K, and Cl concentrations. Monitoring of fluid-electrolyte balance and serum sodium is necessary to avoid any complications such as hypovolemia and dehydration. Moreover, we concord to initiate tolvaptan at low doses and then to increase it gradually according to the fluid-electrolyte balance in order to avoid rapid overcorrection of serum sodium levels, which can lead to severe neurological complications.

Due to the limited data on tolvaptan use in children, little is known about its adverse effects in the pediatric population. In adults, potential adverse effects of tolvaptan include thirst and dry mouth, urinary frequency, fatigue, rapid overcorrection of serum sodium levels, and liver injury (29). Higashi et al. (22) reported the case series of 34 pediatric patients given tolvaptan for congestive heart failure, six of whom experienced thirst and dry mouth, and one patient had a mild increase in liver enzymes. However, in all the reported cases tolvaptan was well-tolerated and is considered to be a safe treatment (21–28).

No side effects have occurred after 6 years of tolvaptan treatment in our patient. However, since there is limited information available regarding tolvaptan use in children and due to the concerns for liver toxicity with long-term use, close monitoring for side effects is warranted.

The strength of our report is the need to inform the scientific community about tolvaptan use in children. The major merits were positive results for this therapy and high applicability when other research designs were difficult to carry out. The major limitations were the inability to generalize and the retrospective design.

In conclusion, we described a rare case of a SAC associated with SIADH in a 3-year-old child. Our experience highlighted the importance of considering SIADH as an etiology for hyponatremia in patients who have undergone neurosurgery.

## Patient Perspective

Treatment of SIADH should start with prevention. Recognition of children at risk can allow tailored IV or fluid therapy and more careful monitoring. In the pediatric age group, tolvaptan can be considered as a useful treatment for chronic hyponatremia due to SIADH, especially in patients who do not respond to other treatment options. In addition, tolvaptan has advantageous aspects compared with other treatment such as fluid liberalization and for treatment compliance. Further data are needed to strengthen its effectiveness and safety.

## Data Availability Statement

The original contributions presented in the study are included in the article/supplementary materials, further inquiries can be directed to the corresponding author/s.

## Ethics Statement

Ethical approval was not provided for this study on human participants because In accordance with the University of Verona and the Institutional Review Board (IRB) policies of the University of Verona, case reports are not considered to be research studies subject to IRB review and are thus exempted from formal IRB approval for publication. We did, however, obtain written consent from the patient for publication of our findings. Written informed consent to participate in this study was provided by the participants' legal guardian/next of kin.

## Author Contributions

RG and MB designed the study. AP and PC collected data. RG, AP, and MB interpreted the data. AP and MB wrote the first draft of the manuscript. RG, PC, MZ, GP, and MB critically reviewed the manuscript for intellectual content. All authors contributed to the article and approved the submitted version.

## Conflict of Interest

The authors declare that the research was conducted in the absence of any commercial or financial relationships that could be construed as a potential conflict of interest.
